# Nephroprotective Effects of *Fraxinus Hookeri Wenz.* Against Renal Toxicity and DNA Oxidative Damages Induced by CCl_4_ in Rats

**DOI:** 10.1002/open.202400515

**Published:** 2025-06-16

**Authors:** Raheela Sarwar, Bashir Ahmad, Ayman M. Al‐Qaaneh, Shumaila Rauf, Laiba Ahmad, Mounir M. Bekhit, Said Hassan, Nadeem Ullah, Gokhan Zengin, Arshad Farid

**Affiliations:** ^1^ Center of Biotechnology and Microbiology University of Peshawar Peshawar Khyber Pakhtunkhwa Pakistan; ^2^ Faculty of Allied Health Sciences Al‐Balqa Applied University (BAU) Al‐Salt 19117 Jordan; ^3^ Department of Pharmaceutical Technology Faculty of Pharmacy Jordan University of Science and Technology (JUST) Irbid 22110 Jordan; ^4^ Department of Pharmacy University of Peshawar Peshawar Khyber Pakhtunkhwa Pakistan; ^5^ Khyber Teaching Hospital Peshawar Peshawar Khyber Pakhtunkhwa Pakistan; ^6^ Department of Pharmaceutics College of Pharmacy King Saud University PO Box 2457 Riyadh 11451 Saudi Arabia; ^7^ Department of Health and Biological Sciences Abasyn University Peshawar Peshawar Khyber Pakhtunkhwa Pakistan; ^8^ Department of Clinical Microbiology Umeå University Umeå Sweden; ^9^ Department of Biology Science Faculty Selcuk University Konya Turkey; ^10^ Gomal Center of Biochemistry and Biotechnology Gomal University D. I. Khan 29050 Pakistan

**Keywords:** CCl_4_, *Fraxinus hookeri*, genotoxicity, histopathology, nephroprotective, nephrotoxicity, renal

## Abstract

Recognizing the therapeutic value of the Genus *Fraxinus* worldwide, this study evaluates the antioxidant potential of *Fraxinus hookeri Wenz.* (*F. hookeri*) against CCl_4_‐induced nephrotoxicity in rats. Forty‐eight rats are randomly allocated into eight groups (six rats each). Antioxidant enzymes, genotoxicity, urine and serum markers, and tissue histopathology are assessed to determine their nephroprotective effects. The Control group remains untreated, while the DMSO group receives vehicle olive oil intraperitoneally and DMSO orally (3 ml/kg). All other groups, except Control and DMSO, are given CCl_4_ (3 ml/kg, i.p., in 30% olive oil) twice weekly for 4 weeks. The CCl_4_ group receives only CCl_4_. The Rutin group receives reference drug Rutin orally (50 mg/kg). MEFH100 and MEFH200 groups are given MEFH at 100 and 200 mg/kg, respectively, and NHFH100 and NHFH200 receive NHFH at the same doses. Rutin and *F. hookeri* treatment effectively (*P* < 0.05) restore urine and serum markers disrupted by CCl_4_. CCl_4_ reduced (*p* < 0.05) antioxidant enzymes (CAT, SOD, POD) and increased TBAR levels and DNA damage, which are reversed by cotreatment with *F. hookeri* and Rutin. Histopathological improvements (*P* < 0.05) are also observed with *F. hookeri.* The results indicate that *F. hookeri* enhances antioxidant defenses, supporting its potential against CCl_4_‐induced nephrotoxicity.

## Graphical abstract

This study evaluates the nephroprotective potential of *Fraxinus hookeri Wenz*. (*F. hookeri*), a medicinal plant of the Oleaceae family, against CCl_4_‐induced oxidative stress. The findings demonstrate its ability to restore antioxidant defenses, renal function markers, and DNA integrity while reducing lipid peroxidation and histopathological damage. *F. hookeri* emerges as a promising candidate for natural therapeutic intervention in oxidative stress‐mediated kidney injury, opening new avenues for future pharmacological and clinical applications.

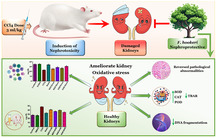

## 1 Introduction

Kidney disease is the 9th leading cause of death, affecting over 15% of US adults, or about 37 million people.^[^ ^1^ ^]^ Diabetes mellitus is the primary cause of chronic kidney disease, followed by hypertension and glomerulonephritis. Other contributing factors include idiopathic causes, often associated with reduced kidney size.^[^ ^2^, ^3^, ^4^ ^]^ Carbon tetrachloride (CCl_4_), widely utilized in metal degreasing and various industrial processes, generates reactive oxygen species (ROS) and depletes antioxidant defenses, leading to oxidative stress in multiple organs, including the kidneys, heart, liver, brain, blood, lungs, and testes. Due to its capacity to induce oxidative damage, CCl_4_ is frequently employed as an experimental model for studying oxidative stress‐related pathologies.^[^ ^5^ ^]^ CCl_4_ can induce severe renal injury in rats by impairing the antioxidant defense system, potentially resulting in nephropathies.^[^ ^6^ ^]^ During biotransformation by cytochrome P450 enzymes, primarily cytochrome P450 2E1 (CYP2E1), CCl_4_ is converted into the highly reactive trichloromethyl radical (•CCl_3_). In the presence of oxygen, this radical can further transform into the more reactive trichloromethyl peroxyl radical (•OOCCl_3_), leading to the initiation of lipid peroxidation.^[^ ^7^ ^]^ These free radicals are implicated in CCl_4_‐induced nephrotoxicity, causing extensive damage to lipids and DNA, along with the accumulation of dysfunctional proteins, leading to severe kidney injuries.^[^ ^8^ ^]^ Traditional medicinal plants are increasingly recognized for their therapeutic potential against various diseases.^[^ ^9^ ^]^

Plant‐derived components and herbal extracts have shown protective properties against oxidative stress caused by chemicals like CCl_4_.^[^ ^10^ ^]^ Medicinal herbs contain essential endogenous antioxidants that protect cells from oxidative damage. Researchers are increasingly investigating the antioxidant properties of medicinal plants to treat oxidative stress‐related diseases. The global demand for medicinal plants has grown due to their perceived therapeutic potential, affordability, and lack of adverse effects.^[^ ^11^ ^]^ Natural antioxidants in plant extracts show promise by scavenging free radicals and mitigating chemically induced damage. Secondary metabolites, such as phenolic compounds, polyphenols, and flavonoids, play a key role in these therapeutic effects.^[^ ^12^ ^]^ The ancient use of plants in medicine has contributed to developing new therapeutic agents.^[^ ^13^ ^]^

*F. hookeri* is a notable member of the Oleaceae family, locally recognized as Toor or Kasunar, and flourishes as a grand deciduous flowering tree. Within Pakistan, it flourishes in Hazara, Azad Kashmir, and Swat while also being widespread across Asia and the Asia‐Pacific, reaching heights between 18 and 30 meters and diameters spanning from 0.6 to 1.0 meters.^[^ ^14^, ^15^ ^]^ Throughout history, *Fraxinus* has been used in folk medicine across the globe to treat a broad range of pharmacological problems.^[^ ^16^ ^]^ The stem bark of *F. hookeri* is brewed into a decoction by local communities, valued for its efficacy in combating pneumonia and typhoid fever.^[^ ^17^ ^]^ Moreover, its bark and leaves are also used to reduce astringency.^[^ ^14^ ^]^ *Fraxinus rhynchophylla* leaf water extract protected male mice against acetaminophen‐induced nephrotoxicity. The stem bark of *Fraxinus xanthoxyloides* is employed to treat trauma, reduce labor pain, and avoid premature birth.^[^ ^18^ ^]^ *Fraxinus micrantha* is known for its efficacy in addressing liver problems, whereas *Fraxinus malacophylla* roots are beneficial against inflammation, malaria, and anti‐rhinitis.^[^ ^19^ ^]^

However, no scientific studies have been conducted on the nephroprotective effects of *F. hookeri.* Thus, this investigation aims to assess the possible nephroprotective ability of organic solvent extracts of *F. hookeri* in renal damage generated by CCl_4_. Serum‐level renal function tests, biochemical tests of urine, and tissue damage, including histopathology, DNA ladder, DNA fragmentation, and antioxidant enzyme assays, were assessed.

## 2 Experimental Section

### 2.1 Collection of Plant

In April 2022, aerial parts of *F. hookeri* were collected from Swat, Khyber Pakhtunkhwa, Pakistan. The taxonomic identification to the species level was carried out using the morphological characteristics of the plants with voucher ID Bot.20323 (PUP), and voucher specimens were deposited in the herbarium of the University of Peshawar. After two weeks of shade drying, all plant parts were chopped and mechanically crushed to a 1 mm mesh size.

### 2.2 Preparation of Methanolic and n‐Hexane Extract

The plant powder (≈1 kg) underwent methanol extraction (2 L) for three weeks with occasional stirring. After filtering through Whatmann No. 45 paper, the extract was concentrated at 40 °C using a low‐pressure vacuum rotary evaporator. The n‐hexane extraction involved adding 2 L n‐hexane to half of the dried methanolic extract, shaking the mixture, and letting it stand for 1 h. Subsequently, concentrating it again under the same conditions as the methanolic extract. For in vivo investigation, both methanolic (highly polar) and n‐hexane (less polar) fractions were examined.^[^ ^20^ ^]^

### 2.3 Purchase of Experimental Animals

Randomly selected forty‐eight (48) Sprague–Dawley rats were purchased from PCSIR (Pakistan Council of Scientific and Industrial Research), Peshawar. The study protocol was approved by the Research Ethical Committee for the University of Peshawar, with Protocol Identification Number 58/Bio‐FLES‐UOP/2022, Ref/no. 907/Bio. All rats weighing 200 ± 50 g were accommodated in polypropylene cages, maintained under standard conditions (23 °C) with availability to clean tap water and a nutritionally balanced diet *ad libitum*.^[^ ^10^ ^]^ The rats were housed in the animal house of the Department of Pharmacy at the University of Peshawar, Pakistan, in compliance with the study strategy authorized by the Institutional Ethical Committee for Animal Care and Experimentation.

### 2.4 Experimental Design

The study allocated forty‐eight rats into eight groups, each comprising six rats, to investigate the nephroprotective effects of the Methanolic Extract of *F. hookeri* (MEFH) and the n‐hexane fraction of *F. hookeri* (NHFH). Plant extracts were administered after 48 h of CCl_4_ treatment twice a week for four (4) weeks **Table** **1**.

**Table 1:** Animal study groups and doses administered orally and intraperitoneally.

Studied groups	Orally given doses	Intraperitoneally given doses
Control	Untreated	
DMSO	3 mL kg^−1^ b.w.	30% olive oil
CCl_4_	–	3 mL kg^−1^ b.w. CCl_4_ in 30% olive oil.
Rutin	50 mg in 3 mL of DMSO	
MEFH_100_	100 mg kg^−1^ b.w.	
MEFH_200_	200 mg kg^−1^ b.w.	
NHFH_100_	100 mg kg^−1^ b.w.	
NHFH_200_	200 mg kg^−1^ b.w.	

### 2.5 Sample Preparation (Blood, Urine, and Kidney Tissue)

After four (4) weeks, animals were anesthetized with chloroform and whole blood was extracted from the heart through cardiac puncture to test oxidative stress. The kidneys were removed after being dissected from the ventral side and then washed with an ice‐cold saline solution. Subsequently, the tissues were divided into two segments: one for DNA damage and enzymatic analysis that was subjected to treatment with liquid nitrogen and preserved at −80 °C, and the second one was promptly stored in formalin solution (10%, PH 7.4) for histopathology. Serum was extracted from blood samples through centrifugation (3000 rpm, 4 °C, 10 min), which was subsequently preserved at −20 °C for further assays.^[^ ^21^ ^]^

#### 2.5.1 Histopathological Studies

Formalin‐fixed kidney tissues were dehydrated with increasing ethanol concentrations (70%, 80%, and 90%) and cleared in xylene. After tissues achieved clarity, they were immersed in molten paraffin wax for embedding (24 h at 56 °C in a hot air oven). Using a microtome, 5 μm sections of embedded tissues were cut, then fixed onto glass slides and stained accordingly for photo microscopy using Hematoxylin and Eosin (H&E). Histopathological changes were evaluated through slide analysis using a 40 × Nikon ECLIPSE E200 microscope magnifications.^[^ ^21^ ^]^

#### 2.5.2 Analysis of Urine Profile

Urine samples were assayed for urinary urea, specific gravity, RBC, Protein, white blood cell (WBC), urobilinogen, creatinine, albumin, creatinine clearance, and PH by using standard adenosine monophosphate (AMP) diagnostic kits (Stattogger Strasse 31b 8045 Graz, Austria). Absorbance measurements of the test specimen and standard were taken, and parameter values were calculated using the kit's formulas.

#### 2.5.3 Biochemical Studies of Serum

Through the use of appropriate commercial diagnostic kits (AMP), kidney function indices, including serum urea, direct bilirubin, total bilirubin, creatinine, urobilinogen, serum nitrite, total protein, albumin, globulin, and creatinine clearance, were assessed following the guidelines supplied by the manufacturer.^[^ ^22^ ^]^

#### 2.5.4 Biochemical Estimation of Renal Antioxidant Activity

To determine the antioxidant enzyme concentration, 10 volumes of Potassium dihydrogen phosphate buffer (100 mM) comprising ethylenediamine tetraacetic acid (EDTA) (1 mM, pH 7.4) were applied to homogenize the renal tissue section (100 mg). Following that, the final mixture was then centrifuged for 30 min (12,000 × g, 4 °C). The collected supernatant was utilized to evaluate antioxidant enzymes, as indicated below.^[^ ^23^ ^]^

#### 2.5.5 Superoxide Dismutase (SOD)

SOD activity was assessed following the method described by Kakkar et al. (1984), with slight modifications.^[^ ^24^ ^]^ Kidney tissue homogenate was prepared in ice‐cold phosphate buffer (50 mM, pH 7.4) and centrifuged at 1500 × g for 10 min to remove debris. The resulting supernatant was centrifuged at 10 000 × g for 15 min to obtain a clear enzyme extract. The reaction mixture consisted of 1.2 mL of sodium pyrophosphate buffer (Na_4_P_2_O_7_, 0.052 mM, pH 7.0), 0.1 mL of phenazine methosulfate (PMS), and 0.3 mL of the enzyme extract. The enzyme reaction was initiated by adding 0.2 mL of Nicotinamide Adenine Dinucleotide (NADH) (780 μM) to the mixture, and the reaction was allowed to proceed for 1 min. The reaction was terminated by adding 1 mL of glacial acetic acid, which facilitated the color development of the reaction product. The absorbance of the resulting chromogen was measured at 560 nm using a UV–visible spectrophotometer. One unit of SOD activity was defined as the amount of enzyme required to inhibit the rate of NADH oxidation by 50% under the assay conditions. The enzyme activity was expressed as units per milligram of protein (U/mg protein). Appropriate controls without the enzyme extract were included to account for non‐enzymatic reactions.

#### 2.5.6 Catalase Assay (CAT)

CAT activity was measured using a slightly modified version of the method described by Chance and Maehly (1955).^[^ ^25^ ^]^ Briefly, the enzyme extract (0.1 mL) was added to 2.5 mL of phosphate buffer (50 mM, pH 5.0) in a cuvette. The reaction was initiated by adding 0.4 mL of hydrogen peroxide (H_2_O_2_, 5.9 mM) to the mixture. The decrease in absorbance at 240 nm, due to the decomposition of H_2_O_2_, was monitored for 1 min using a UV–visible spectrophotometer. One unit of CAT activity was defined as the amount of enzyme required to decompose 0.01 units of H_2_O_2_ per minute under the assay conditions. The enzyme activity was expressed as units per milligram of protein (U/mg protein), with appropriate controls included to correct for non‐enzymatic degradation of H_2_O_2_.

#### 2.5.7 Peroxidase Assay (POD)

The POD activity was determined using a modified version of the method described by Calberg and Manervik (1975).^[^ ^26^ ^]^ The reaction mixture consisted of 2.5 mL of phosphate buffer (50 mM, pH 5.0), 0.1 mL of guaiacol (20 mM), 0.3 mL of hydrogen peroxide (H_2_O_2_, 40 mM), and 0.1 mL of enzyme extract. The reaction was initiated by adding the enzyme extract to the mixture, and the increase in absorbance at 470 nm, due to the oxidation of guaiacol to tetra‐guaiacol, was recorded for 1 min using a UV–visible spectrophotometer. One unit of POD activity was defined as the amount of enzyme required to produce a change in absorbance of 0.01 units per minute under the assay conditions. The enzyme activity was expressed as units per milligram of protein (U/mg protein). Appropriate controls without the enzyme extract were included to account for any non‐enzymatic oxidation of guaiacol.

#### 2.5.8 Estimation of Lipid Peroxidation

Lipid peroxidation was estimated using a modified technique developed by Iqbal et al.^[^ ^27^ ^]^ In a shaking water bath, the reaction mixture was incubated (1 h, 37.8 °C). It contained 100 mM ferric chloride (0.02 mL), homogenate sample (0.2 mL), 100 mM ascorbic acid (0.2 mL), and 0.1 m phosphate buffer (pH 7.4, 0.58 mL). To stop the reaction, 10% of C_2_HCl_3_O_2_ (1.0 mL) was added. Subsequently, for 20 min, the tubes were boiled after adding 1.0 mL of C_4_H_4_N_2_O_2_S (0.67%). The tubes underwent centrifugation (2500 × g, 10 min) after being allowed to cool in a crushed ice bath. Relative to a reagent blank, the optical density of the supernatant (535 nm) was measured to quantify the amount of thiobarbituric acid reactive substances (TBARS) in each specimen. At 37.8 °C, the findings were displayed as nM TBARS/min/mg tissue when applied as the molar extinction coefficient.

#### 2.5.9 Genotoxicity Studies

The following tests were carried out in the genotoxicity investigation.

#### 2.5.10 Percent DNA Fragmentation Assay

In conducting percent DNA fragmentation analysis in tissue extract, the approach described by Wu et al. (2005)^[^ ^28^, ^29^ ^]^ was followed. In a tris‐triton EDTA lysis buffer pH 8.0, a 100 mg kidney tissue specimen was homogenized. After centrifuging (4 °C, 200 × g, 10 min), a 0.1 mL aliquot of each homogenate (marked as B) and then the supernatant (marked as S) was collected. S tubes were centrifuged (20,000 × g, 4 °C, 10 min) to extract the intact chromatin, which was designated as T. Trichloroacetic acid (TCA) (0.1 mL, 25%) was added to each tube (S, B, and T), incubated overnight to precipitate DNA, and centrifuged.^[^ ^30^ ^]^ Every pellet of precipitate DNA was subjected to 160 μl of 5% TCA and heated at 90 °C (15 min), followed by the 320 μl addition of diphenylamine (DPA) solution. After incubation, optical density was quantified at 600 nm using a spectrophotometer. To express the results, the following formula was applied to the percentage of fragmented DNA;^[^ ^31^ ^]^

Fragmented DNA % = T × 100/T + B

#### 2.5.11 Isolation of DNA and Ladder Assay

The DNA ladder assay was used to estimate DNA damage and to identify the size of damaged DNA fragments. Using Wu et al.'s techniques, DNA was extracted.^[^ ^28^, ^29^ ^]^ In the gel electrophoresis setup (1.5% agarose gel, 1.0 μg ml^−1^ ethidium bromide), 5 μg of extracted DNA alongside a DNA ladder was separately loaded and run for 45 min (100 V). It was then examined and photographed on camera using a digital gel document system.

### 2.6 Statistical Analysis

The data, expressed as Mean ± SD, were checked for statistical analysis through analysis of variance followed by Student T tests for multiple variance, analysis to test the homogeneity of variance, considering a *P* value < 0.05 as statistically significant.

## 3 Results

### 3.1 Effect of *F. hookeri* on CCl_4_‐Induced Nephrotoxicity in Rats

The following section presents the nephroprotective results of *F. hookeri*'s various fractions and Rutin against renal oxidative damage induced by CCl_4_.

### 3.2 Effects on Renal Histopathology

**Figure 1:**
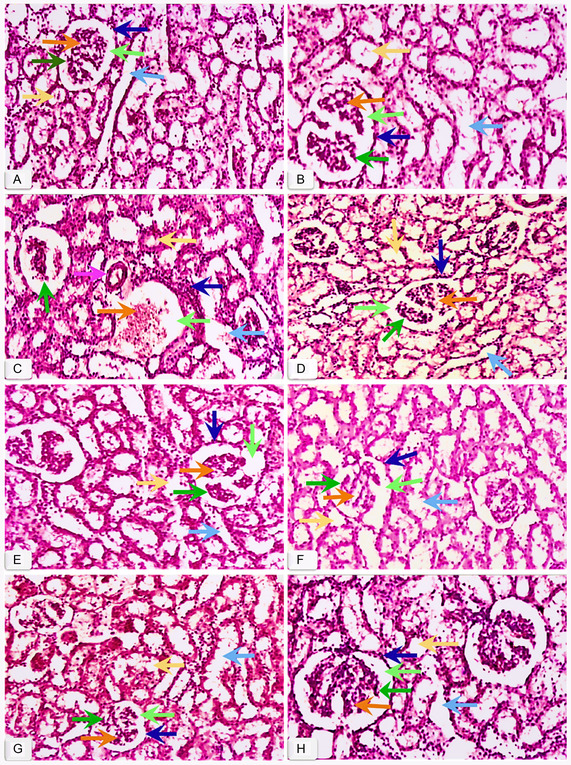
Microscopic images of H&E‐stained kidney sections representing all research groups. A) Control group, B) DMSO + Olive oil, C) CCl_4_, D) Rutin + CCl_4_, and *F. hookeri* extracts Groups E) MEFH_100_ + CCl_4_, F) MEFH_200_ + CCl_4_, G) NHFH_100_ + CCl_4_, and H) NHFH_200_ + CCl_4_. The magnification for all other groups is 40×. Glomerulus (dark green arrow), Mesangium (orange arrow), Bowman's capsule (dark blue arrow), Bowman's space (light green arrow), Distal tubules (light blue arrow), Proximal tubules (yellow arrow), Thick wall vein (pink arrow).

**Figure** **1** presents the renal histological structure from various groups. The histological representation of the Control (A) and dimethyl sulfoxide (DMSO) (B) groups in Figure 1 exhibits the normal preserved structural integrity of renal tubules and Bowman's capsule. Rats treated with CCl_4_ (Figure 1C) had extensive kidney damage in both the cortical and medullary regions. The pathophysiology of CCl_4_ was characterized by infiltration of interstitial inflammatory cells, degenerated renal tubules, obstruction of the lumen with a foamy appearance in the tubular epithelial cells, and remarkable glomerular space expansion with damaged Bowman's capsule and collecting ducts. Figure 1 clearly illustrates the repairing effects observed in groups treated with different fractions of *F. hookeri*. The histological findings of MEFH_100_ (E) and NHFH_100_ (G) indicated that the majority of glomeruli and Bowman's capsules displayed nearly normal appearances, with only a few showing signs of degeneration, while tubules demonstrated widened lumens with prominent nuclei, effectively preventing interstitial edema. The most significant protection among all fractions was observed with MEFH_200_ (F) and NHFH_200_ (H), resulting in a renal structure that closely resembled the Control and DMSO groups. Moreover, the Rutin group exhibited a marked decrease in renal pathology compared to the CCl_4_‐treated group (Figure 1D).

### 3.3 Effects on Urine Profile of Rat

**Figure 2:**
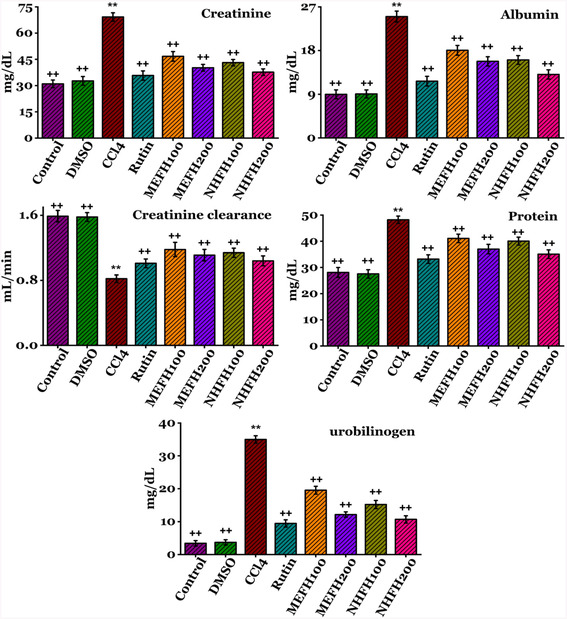
Evaluation of the nephroprotective effects of *F. hookeri* different fractions on urine profile levels, including urobilinogen, creatinine, albumin, creatinine clearance, and protein in rats, with Mean ± SD shown for every group (*n* = 6). ++ indicate significance from the CCl_4_ group at *P* < 0.05 and probability level is *P* < 0.01. ** indicate significance from the Control group at *P* < 0.05 and probability level is *P* < 0.01.

**Figure 3:**
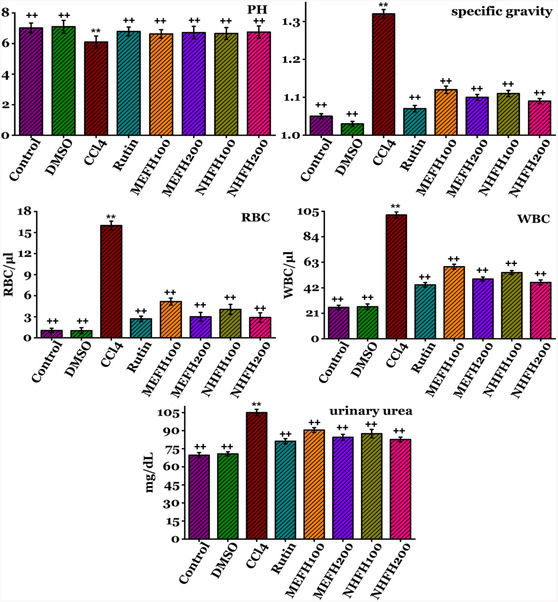
Evaluation of the nephroprotective effects of *F. hookeri* different fractions on urine profile levels, including urinary urea, specific gravity, RBC, PH, and WBC in rats, with Mean ± SD shown for every group (*n* = 6). ++ indicate significance from the CCl_4_ group at *P* < 0.05 and probability level is *P* < 0.01. ** indicate significance from the Control group at *P* < 0.05 and probability level is *P* < 0.01.

The protective effects of *F. hookeri* against CCl_4_ administration on renal functions are outlined in **Figure** **2** (Table S1, Supporting Information) and **Figure** **3** (Table S2, Supporting Information). Significant increases (*P* < 0.05) in urinary urea (105 ± 2.61 mg dL^−1^), specific gravity (16 ± 0.61), RBC (16 ± 0.61), Protein (48.2 ± 1.41 mg dL^−1^), WBC (102 ± 2.55), urobilinogen (35 ± 1.13 mg dL^−1^), creatinine (69.24 ± 2.36 mg dL^−1^), and albumin (25 ± 1.13 mg dL^−1^) levels were observed after CCl_4_ administration, along with a significant decrease (*P* < 0.05) in both creatinine clearance (0.82 ± 0.04 mL min^−1^) and PH (6.1 ± 0.39) in contrast to the DMSO and Control groups. Rats treated with different fractions of *F. hookeri* and Rutin exhibited a reversal of CCl_4_ toxicity, with a significant recovery of the parameters mentioned above, bringing them closer to those observed in the Control group particularly by higher dose 200 mg kg^−1^ of MEFH_200_; urinary urea (84.5 ± 2.40 mg dL^−1^), specific gravity (1.1 ± 0.007), RBC (3 ± 0.61), Protein (37.01 ± 1.81 mg dL^−1^), WBC (49.2 ± 1.63), urobilinogen (12.14 ± 0.81 mg dL^−1^), creatinine (40.2 ± 1.88 mg dL^−1^), albumin (15.77 ± 0.95 mg dL^−1^), creatinine clearance (1.11 ±0.07 mL min^−1^), and PH (6.72 ± 0.40), and NHFH_200_; urinary urea (82.6 ± 1.93 mg dL^−1^), specific gravity (1.09 ± 0.006), RBC (2.9 ± 0.66), Protein (35.09 ± 1.63 mg/dL), WBC (46.34 ± 2.07), urobilinogen (10.69 ± 1.09 mg dL^−1^), creatinine (37.68 ± 1.88 mg dL^−1^), albumin (13.09 ± 0.95 mg dL^−1^), creatinine clearance (1.04 ±0.06 mL min^−1^), and PH (6.75 ± 0.40). The supplementary data provides details on the results for all other groups.

### 3.4 Effects on Serum Profile of Rats

**Figure 4:**
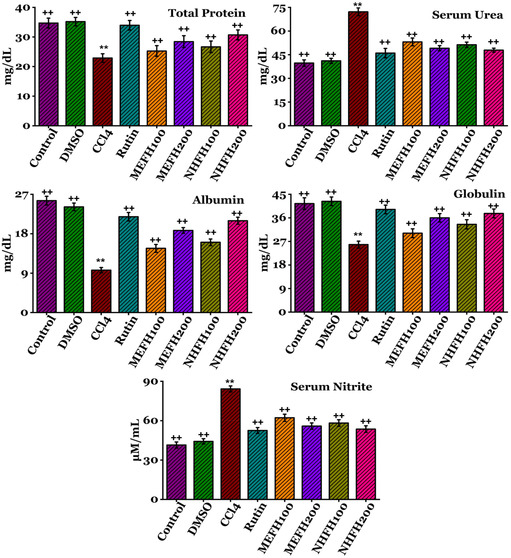
In rats, the nephroprotective effects of *F. hookeri* different fractions on serum profile levels, including serum protein, globulin, serum urea, albumin, and serum nitrite, with Mean ± SD shown for every group (*n* = 6). ++ indicate significance from the CCl_4_ group at *P* < 0.05 and probability level is *P* < 0.01. ** indicate significance from the Control group at *P* < 0.05 and probability level is *P* < 0.01.

**Figure 5:**
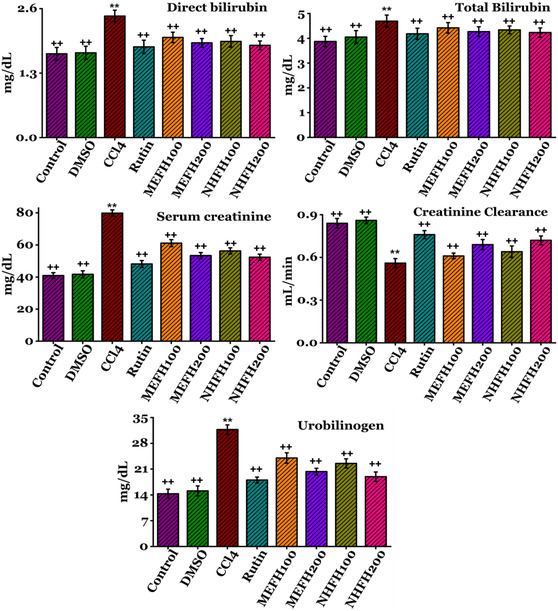
Nephroprotective effects of *F. hookeri* different fractions on serum profile levels, including direct bilirubin, total bilirubin, creatinine, urobilinogen, and creatinine clearance in rats, with Mean ± SD shown for every group (*n* = 6). ++ indicate significance from the CCl_4_ group at *P* < 0.05 and probability level is *P* < 0.01. ** indicate significance from the Control group at *P* < 0.05 and probability level is *P* < 0.01.

The impacts of *F. hookeri*'s various fractions on the renal serum profile are documented in **Figure** **4** (Table S3, Supporting Information) and **Figure** **5** (Table S4, Supporting Information). Upon administration of CCl_4_ to rats, there was a significant (*P* < 0.05) rise in the concentrations of serum urea (72.2 ± 2.44 mg dL^−1^), direct bilirubin (2.45 ± 0.11 mg dL^−1^), total bilirubin (4.69 ± 0.24 mg dL^−1^), creatinine (79.87 ± 1.98 mg dL^−1^), urobilinogen (31.74 ± 1.27 mg dL^−1^), serum nitrite (84.18 ± 2.25 μM mL^−1^), while decrease (*P* < 0.05) in total protein (22.88 ± 1.45 mg dL^−1^), albumin (9.69 ± 0.60 mg dL^−1^), globulin (25.78 ± 1.32 mg dL^−1^), and creatinine clearance (0.56 ± 0.03 mL min^−1^) in contrast to the DMSO and Control groups. The toxicity induced by CCl_4_ in the serum profile of the kidney was effectively ameliorated by the administration of various fractions of *F. hookeri*, leading to a significant (*P* < 0.05) restoration to nearly control levels. Rutin was selected as the reference drug to decrease nephrotoxicity. The MEFH_200_ (serum urea (49.09 ± 1.63 mg dL^−1^), direct bilirubin (1.91 ± 0.08 mg dL^−1^), total bilirubin (4.27 ± 0.19 mg dL^−1^), creatinine (53.34 ± 1.89 mg dL^−1^), urobilinogen (20.33 ± 0.90 mg dL^−1^), serum nitrite (55.89 ± 2.36 μM mL^−1^), total protein (28.42 ± 2.002 mg dL^−1^), albumin (18.76 ± 0.65 mg dL^−1^), globulin (36.02 ± 1.60 mg dL^−1^), and creatinine clearance (0.69 ± 0.03 mL min^−1^)), and NHFH_200_ (serum urea (47.87 ± 1.26 mg dL^−1^), direct bilirubin (1.86 ± 0.08 mg dL^−1^), total bilirubin (4.23 ± 0.18 mg dL^−1^), creatinine (52.33 ± 1.92 mg dL^−1^), urobilinogen (18.96 ± 1.28 mg dL^−1^), serum nitrite (53.6 ± 2.51 μM mL^−1^), total protein (30.69 ± 1.69 mg dL^−1^), albumin (20.98 ± 0.79 mg dL^−1^), globulin (37.69 ± 1.68 mg dL^−1^), and creatinine clearance (0.72 ± 0.03 mL min^−1^)) demonstrated relatively more remarkable improvement compared to the other fractions. The results for all other groups are detailed in the supplementary data.

### 3.5 Effects on Renal Enzymatic Antioxidant Levels

**Figure 6:**
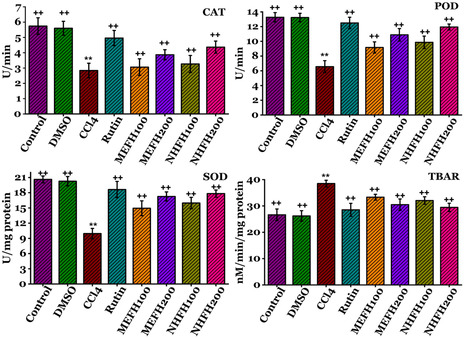
Evaluation of the nephroprotective effects of *F. hookeri* different fractions on the enzymatic antioxidant levels in rats, with Mean ± SD shown for every group (*n* = 6). ++ indicate significance from the CCl_4_ group at *P* < 0.05 and probability level is *P* < 0.01. ** indicate significance from the Control group at *P* < 0.05 and probability level is *P* < 0.01.

In order to determine the toxicity of CCl_4_ and the protective effects of *F. hookeri* on rat kidneys, the assessment of antioxidative enzymes was conducted in this work, as shown in **Figure** **6** (Table S5, Supporting Information). Rats administered CCl_4_ showed markedly lower levels (*P* < 0.05) of antioxidative status as measured by renal CAT (2.84 ± 0.47 U/min), POD (6.56 ± 0.80 U min^−1^), and SOD (9.93 ± 1.02 U mg^−1^ protein) levels; also, there was a substantial increase (*P* < 0.05) in TBAR (38.50 ± 1.25 nM min mg^−1^ protein) compared to the Control and DMSO groups. While coadministration of Rutin and *F. hookeri* fractions with CCl_4_ decreased the activity of elevated TBARS, it increased the activity of suppressed enzymes. Data indicates non‐significant (*P* > 0.05) variations as compared to the DMSO and Control groups. The MEFH_200_ and NHFH_200_ were responsible for the greatest recovery effects i.e., MEFH_200_; CAT (3.86 ± 0.32 U min^−1^), POD (10.88 ± 0.85 U min^−1^), SOD (17.26 ± 0.89 U mg^−1^ protein), and TBAR (30.50 ± 2.14 nM min mg^−1^ protein), and NHFH_200_; CAT (4.36 ± 0.39 U min^−1^), POD (11.95 ± 0.4 U min^−1^), SOD (17.83 ± 0.70 U mg^−1^ protein), and TBAR (29.46 ± 1.56 nM min mg^−1^ protein). Supplementary data contains the results for all other groups.

### 3.6 Genotoxicity Studies

#### 3.6.1 Effects on Renal DNA Damages (DNA Ladder Assay)

**Figure 7:**
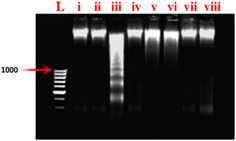
Agarose gel displays the nephroprotective properties of different *F. hookeri* fractions and the DNA damage caused by CCl_4_. From left to right lanes (L) low molecular weight marker, (i) Control, (ii) DMSO, (iii) CCl_4_, (iV) Rutin, and *F. hookeri* extracts Groups: (V) MEFH_100_ + CCl_4_, (Vi) NHFH_100_ + CCl_4_, (Vii) MEFH_200_ + CCl_4_, and (Viii) NHFH_200_ + CCl_4_.

**Figure** **7** shows the DNA banding patterns. In contrast to Control and DMSO‐treated rats, the renal tissues of CCl_4_‐intoxicated rats showed clear signs of DNA fragmentation. Compared to the group administered with CCl_4_ to the one treated with MEFH_100_ and NHFH_100_, there was an improvement in DNA fragmentation. The kidney tissues treated with Rutin, MEFH_200_, and NHFH_200_ showed intact genomic DNA banding patterns in the DNA ladder experiment, suggesting possible protective effects against CCl_4_‐induced DNA damage.

#### 3.6.2 Percent DNA Fragmentation Assay

**Figure 8:**
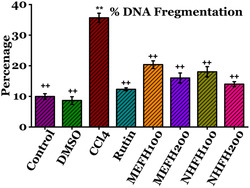
Evaluation of the nephroprotective effects of *F. hookeri* by comparing several renal DNA fragmentation percentage levels across various study groups of rats. Mean ± SD is displayed for each group (*n* = 6). ++ indicate significance from the CCl_4_ group at *P* < 0.05 and probability level is *P* < 0.01. ** indicate significance from the Control group at *P* < 0.05 and probability level is *P* < 0.01.

Damage to the DNA in the rat renal tissue occurs when free radicals created by CCl_4_ interact with DNA to form adducts. Rats given CCl_4_ showed a significant increase (*P* < 0.05) in DNA fragmentation (35.66 ± 1.49%) compared to the Control group (9.95 ± 0.91%). Conversely, as **Figure** **8** (Table S5, Supporting Information) illustrates, the groups of rats given Rutin and *F. hookeri* different extracts, primarily MEFH_200_ (15.99 ± 1.62) and NHFH_200_ (13.98 ± 0.81%), had significantly reduced DNA fragmentation.

## 4 Discussion

Through the annals of history and across diverse cultures, natural products have consistently served as an immensely valuable source of medicinal compounds, offering a plethora of structural frameworks to guide the ongoing journey of drug development and exploration.^[^ ^32^ ^]^ Medicinal plants have been valued as a traditional healing resource, providing a wide array of herbal remedies and botanical compounds that have been instrumental in developing numerous modern pharmaceuticals.^[^ ^33^ ^]^ Phytochemicals exhibit significant free radical scavenging properties, positioning them as superior antioxidants from plant sources.^[^ ^3^ ^]^

In this in vivo research, *F. hookeri*'s methanol and n‐hexane fractions emerge as a protective agent against nephrotoxicity induced by CCl_4_. CCl_4_ is hepatotoxic and nephrotoxic, causing oxidative damage and inflammation through excessive ROS generation.^[^ ^22^ ^]^ In rats, exposure to CCl_4_ triggers nephrotoxicity through the action of free radicals, which is a matter of considerable health concern.^[^ ^34^ ^]^ Kidneys regulate vital chemicals, which are crucial for eliminating waste. It demonstrates a severe sensitivity to metabolites produced by several toxic substances, as well as xenobiotics. Because natural antioxidants are considered safe substitutes with no adverse effects, interest in antioxidant‐rich natural therapies has increased in response to concerns about pesticide exposure.^[^ ^35^ ^]^ Our research highlights *F. hookeri* as a potential treatment to reduce liver oxidative injuries, offering a nutritional alternative to pharmaceutical approaches for nephrotoxicity.

In the current study, rats treated with CCl_4_ had extensive and subcellular kidney damage in both the cortical and medullary regions, as seen in histopathological examination (Figure 1C). The pathophysiology of CCl_4_ was characterized by fibrosis, disruption of kidney Bowman's capsule, collecting ducts, glomerular and glomeruli injuries and moderate to severe necrosis and tubule‐interstitial alterations similar to the histological changes observed by Dogukan et al.^[^ ^36^ ^]^ in rat renal tissue after prolonged CCl_4_ treatment. These changes may result from nephron overloading, leading to renal dysfunction or tissue destruction due to free radical generation during CCl_4_ metabolism. Different fractions of *F. hookeri* (Figure 1E–H) showed the ability to restore the histological structure of the kidneys back to control levels, signifying the reversal of nephrotoxicity caused by CCl_4_. The most significant protection among all fractions was observed with MEFH_200_ and NHFH_200_, resulting in a renal structure that closely resembled normal. *F. hookeri* extracts suppress CCl_4_‐induced nephrotoxicity through the antioxidative and free radical scavenging properties. Our findings align with research showing that plant derivatives counteract CCl_4_ toxicity and restore normal function, e.g., the effects of *Phyllanthus amarus* ^[^ ^37^ ^]^ and methanol extract of *Artemisia brevifolia* ^[^ ^38^ ^]^ as therapeutic agents against CCl_4_‐induced hepatotoxicity and nephrotoxicity in rats. Moreover, the Rutin group exhibited a marked decrease in renal pathology compared to the CCl_4_‐treated group.

Urinalysis yields critical information concerning kidney function and health.^[^ ^39^ ^]^ The protective effects of *F. hookeri* against CCl_4_ administration on renal functions are outlined in Figure 2 and 3. Significant increases (*P* < 0.05) in urinary urea, specific gravity, RBC, protein, WBC, urobilinogen, creatinine, and albumin levels were observed after CCl_4_ administration, along with a significant decrease (*P* < 0.05) in both creatinine clearance and PH in contrast to the DMSO and Control groups. Elevated RBCs and WBCs in urine can increase specific gravity, potentially indicating kidney damage. Urine‐specific gravity reflects solute concentration and kidney function, with elevated levels suggesting dehydration, renal artery tissues, and reduced blood flow.^[^ ^40^ ^]^ The study shows that CCl_4_ exposure in rats leads to proteinuria and hematuria, indicating oxidative stress, and metabolic kidney dysfunction.^[^ ^41^ ^]^ Normally, urobilinogen is absent in urine, but elevated levels in CCl_4_‐treated rats suggest kidney damage.^[^ ^42^ ^]^ Rats treated with different fractions of *F. hookeri* and Rutin exhibited a reversal of CCl_4_ toxicity, with a significant recovery of the parameters mentioned above, bringing them closer to those observed in the Control group, particularly the protective effect was more evident with a higher dose of n‐hexane (NHFH_200_) and methanol (MEFH_200_) fraction. The antioxidant properties of *F. hookeri* extracts likely contribute to inhibiting renal damage. Phytochemicals, highly effective free radical scavengers, are considered superior plant‐based antioxidants.^[^ ^43^ ^]^ This study aligns with prior research on the extract of the medicinal plant *Petroselinum crispum* protection against CCl_4_‐induced nephrotoxicity in rats.^[^ ^44^ ^]^

Additionally, CCl_4_ causes significant modifications in the serum profile. Upon administration of CCl_4_ to rats, there was a significant (*P* < 0.05) rise in the concentrations of serum urea, direct bilirubin, total bilirubin, creatinine, urobilinogen, serum nitrite, while a decrease (*P* < 0.05) in total protein, albumin, globulin, and creatinine clearance in contrast to the DMSO and Control groups (Figure 4 and 5). Pathological transformations reveal the potential renal damage caused by CCl_4_ treatment. This study suggests that the elevated serum creatinine, alongside reduced protein and albumin levels, are likely consequences of the damaged nephron structure and proteinuria contributing to kidney dysfunction.^[^ ^21^ ^]^ These observations are further in agreement with the findings that renal tissue damages revert to normal and stabilization of the endoplasmic reticulum, leading to increased synthesized protein, damaged by CCl_4_.^[^ ^45^, ^46^ ^]^ CCl_4_ intoxication generates free radicals in multiple organs, including the kidneys, liver, lungs, brain, and blood.^[^ ^47^, ^48^ ^]^ However, the toxicity induced by CCl_4_ in the serum profile of the kidney was effectively ameliorated by administering various fractions of *F. hookeri*, leading to a significant (*P* < 0.05) restoration to nearly control levels. Reduction in serum creatinine and urea concentrations of the sample‐treated groups enhances a contributory mechanism of lowered oxidative stress. The urea and serum creatinine are the final compounds of purine that can modify the glomerular filtration rate. The alteration of the glomerular filtration rate increases the serum creatinine and urea levels associated with renal damage.^[^ ^45^ ^]^ Rutin was selected as the reference drug to decrease nephrotoxicity. MEFH_200_ and NHFH_200_ demonstrated relatively greater improvement compared to the other fractions that effectively reduced CCl_4_ effects, normalizing creatinine, urea, serum albumin, and total protein levels. The outcomes of this investigation support previous findings^[^ ^22^ ^]^ regarding the preventive role of medicinal plants against CCl_4_‐induced nephrotoxicity in rats.

The significant generation of free radicals in the kidney results in elevated lipid peroxidation (TBARS), H_2_O_2_, and decreased activity of antioxidant enzymes. A higher concentration of TBARS and decreased levels of antioxidant enzymes indicated significant oxidative damage to the renal tissues.^[^ ^49^ ^]^ In this research, rats administered CCl_4_ showed markedly lower levels (*P* < 0.05) of antioxidative status as measured by renal CAT, POD, and SOD levels; also, in the meantime, there was a substantial increase (*P* < 0.05) in TBAR compared to the Control and DMSO groups (Figure 6) in renal samples, implicating the presence of oxidative tissue damage. Since the antioxidant defense mechanisms are necessary to eliminate free radicals, they are extremely significant in protecting biological sites. The decreased activities of primary antioxidants, CAT, and SOD, may result from the accumulation of ROS.^[^ ^50^ ^]^ The role of CAT is significant in the conversion of H_2_O_2_ to water, whereas SOD facilitates the conversion of H_2_O_2_ into oxygen. This notion is supported by evidence that SOD activity can be inhibited by H_2_O_2_ treatment. Notably, elevated H_2_O_2_ levels were observed in renal tissues in this study. Assessing the activity levels of these enzymes provides an effective indirect measure of the pro‐oxidant/antioxidant balance in tissues.^[^ ^50^, ^51^ ^]^ Probably, coadministration of Rutin and *F. hookeri* fractions with CCl_4_ ameliorated functional deterioration and oxidative damage. *F. hookeri* acts as an antioxidant agent that decreases the activity of elevated TBARS, whereas it increases the activity of suppressed enzymes. Data indicates non‐significant (*P* > 0.05) variations compared to the DMSO and Control groups. MEFH_200_ and NHFH_200_ were responsible for the greatest recovery effects. The protective benefits of *F. hookeri* against CCl_4_‐induced kidney damage may be attributed to the presence of polyphenolic and other antioxidant ingredients. The outcomes of investigations on oxidative enzymatic reactions align with the findings of studies on serological and urine reactions. This study's enzymatic, serum, and urine findings are consistent with earlier research, highlighting the protective effects of *linalool* against renal and hepatic damage caused by CCl_4_.^[^ ^52^ ^]^

Rats treated with CCl_4_ experienced DNA damage, which broke DNA strands in their kidney tissues.^[^ ^53^ ^]^ CCl_4_ treatment induces DNA adducts, genetic mutations, strand breaks, and chromosomal alterations as a defense against peroxy radicals generated by CCl_4_. DNA strand breaks are particularly significant, leading to mutations like deletions and translocations during error‐prone or inadequate repair. If unrepaired, extensive strand breaks may result in cell death and trigger compensatory cell regeneration.^[^ ^51^, ^54^ ^]^ The DNA banding patterns are shown in Figure 7. In the current investigation, in contrast to Control or DMSO‐treated rats, the renal tissues of CCl_4_‐intoxicated rats showed clear signs of DNA fragmentation (Figure 8). Comparing the group administered with CCl_4_ to the one treated with MEFH_100_ and NHFH_100_, there was an improvement in DNA fragmentation. Its protective effects may result from counteracting CCl_4_‐induced damage through modulation of metabolic cycles, proteasomal degradation, and DNA repair. These protective mechanisms likely involve antioxidant activity, regulation of signal transduction, gene expression, and effective participation in metabolic pathways. The kidney tissues treated with Rutin, MEFH_200_, and NHFH_200_ showed intact genomic DNA banding patterns in the DNA ladder experiment, suggesting possible protective effects against CCl_4_‐induced DNA damage. Consistent findings were reported in a related investigation when assessing the protective consequence of *Carissa opaca* fruits against nephrotoxicity induced by CCl_4_.^[^ ^55^ ^]^

## 5 Conclusions

The study investigated the nephroprotective role of *F. hookeri* at different levels. To sum up, our research clearly demonstrated that *F. hookeri* has the potential to alleviate renal damage caused by CCl_4_, potentially restoring antioxidant functions, DNA integrity, urine and serum compositions, lipid peroxidation levels, and histological structure of renal tissues. These characteristics explain the plant's medicinal efficacy. The Rutin used as a reference drug, and the MEFH_200_ and NHFH_200_ showed greater improvement than other fractions in reducing CCl_4_ effects, signifying that a higher quantity of *F. hookeri* is more therapeutic. Our study provides evidence for the antioxidant efficacy of *F. hookeri*, suggesting its potential utility in the fields of renal health sciences and pharmaceutical development. Future research should explore the bioactive compounds responsible for these protective effects through advanced phytochemical analyzes. Additionally, molecular studies could further elucidate the underlying mechanisms of action, while clinical trials may validate the nutraceutical potential of *F. hookeri* for developing nephroprotective therapeutics.

## Conflict of Interest

The authors declare no conflicts of interest. The funders had no role in the design of the study; in the collection, analyzes, or interpretation of data; in the writing of the manuscript; or in the decision to publish the results.

## Author Contributions

**Raheela Sarwar:** Conceptualization, Methodology, Software, Investigation, Data curation, Writing—original draft preparation, Writing—review and editing. **Bashir Ahmad:** Methodology, Project administration. **Ayman M. Al‐Qaaneh:** Supervision, Funding acquisition. **Shumaila Rauf:** Formal analysis, Investigation, Data curation, Writing—original draft preparation. **Laiba Ahmad:** Validation. **Said Hassan:** Software, Supervision, Project administration. All authors have read and agreed to the published version of the manuscript.

## Supporting information

Supplementary Material

## Data Availability

No data from any repository has been used in Manuscript “Nephroprotective effects of *Fraxinus hookeri Wenz*. against renal toxicity induced by CCl_4_ and DNA oxidative damages in rats” whereas the existing data is already presented originally in the form of regular tables and figures in this manuscript.
