# Supplementary material for: Nephroprotective Effects of Fraxinus Hookeri Wenz. Against Renal Toxicity and DNA Oxidative Damages Induced by CCl4 in Rats
Source: ChemistryOpen. 2025 Jun 16;14(8):e202400515. doi: 10.1002/open.202400515 (PMC12368880; doi:10.1002/open.202400515)
Supplement: Supplementary file 1 — Supplementary Material [file OPEN-14-e202400515-s001.pdf]

Supporting Information (SI)

Table S1: Evaluation of the nephroprotective effects of *Fraxinus hookeri* different fractions on urine profile levels including urobilinogen, creatinine, albumin, creatinine clearance, and protein in rats, with Mean ± SD shown for every group (n = 6). ++ indicate significance from the CCl_4 group at P < 0.05 and probability level is P < 0.01. ** indicate significance from the Control group at P < 0.05 and probability level is P < 0.01.

Groups	Creatinine (mg/dL)	Creatinine clearance (mL/min)	Albumin (mg/dL)	urobilinogen (mg/dL)	Protein (mg/dL)
**Control**	$31±2.16^{++}$	$1.59±0.07^{++}$	$8.98±0.90^{++}$	$3.4±0.83^{++}$	$28.1±1.89^{++}$
**DMSO**	$32.7±2.48^{++}$	$1.58±0.05^{++}$	$9.07±0.81^{++}$	$3.7±0.79^{++}$	$27.54±1.61^{++}$
**CCl4**	$69.24±2.36^{**}$	$0.82±0.04^{**}$	$25±1.13^{**}$	$35±1.13^{**}$	$48.2±1.41^{**}$
**Rutin**	$35.75±2.64^{++}$	$1.01±0.05^{++}$	$11.69±1.00^{++}$	$9.45±1.09^{++}$	$33.2±1.63^{++}$
**$MEFH_{100}$**	$46.7±2.73^{++}$	$1.18±0.08^{++}$	$18.05±1.00^{++}$	$19.55±1.19^{++}$	$41.1±1.63^{++}$
**$MEFH_{200}$**	$40.2±1.88^{++}$	$1.11±0.07^{++}$	$15.77±0.95^{++}$	$12.14±0.81^{++}$	$37.01±1.81^{++}$
**$NHFH_{100}$**	$43.1±1.82^{++}$	$1.14±0.05^{++}$	$16.07±0.89^{++}$	$15.2±1.23^{++}$	$40.05±1.51^{++}$
**$NHFH_{200}$**	$37.68±1.88^{++}$	$1.04±0.06^{++}$	$13.09±0.95^{++}$	$10.69±1.09^{++}$	$35.09±1.63^{++}$

Table S2: Evaluation of the nephroprotective effects of *Fraxinus hookeri* different fractions on urine profile levels including urinary urea, specific gravity, RBC, PH, and WBC in rats, with Mean ± SD shown for every group (n = 6). ++ indicate significance from the CCl_4 group at $P < 0.05$ and probability level is $P < 0.01$. ** indicate significance from the Control group at $P < 0.05$ and probability level is $P < 0.01$.

Groups	PH	specific gravity	RBC/µl	WBC/µl	urinary urea (mg/dL)
**Control**	$7.02±0.31^{++}$	$1.05±0.006^{++}$	$1.04±0.31^{++}$	$25.8±1.76^{++}$	$69.6±2.14^{++}$
**DMSO**	$7.1±0.41^{++}$	$1.03±0.006^{++}$	$1.02±0.41^{++}$	$26.4±2.29^{++}$	$70.7±1.74^{++}$
**CCl4**	$6.1±0.39^{**}$	$1.32±0.01^{**}$	$16±0.61^{**}$	$102±2.55^{**}$	$105±2.61^{**}$
**Rutin**	$6.79±0.28^{++}$	$1.07±0.008^{++}$	$2.7±0.38^{++}$	$44.4±1.83^{++}$	$81.2±2.16^{++}$
**$MEFH_{100}$**	$6.63±0.27^{++}$	$1.12±0.009^{++}$	$5.16±0.49^{++}$	$59.4±1.97^{++}$	$90.5±1.99^{++}$
**$MEFH_{200}$**	$6.72±0.40^{++}$	$1.1±0.007^{++}$	$3±0.61^{++}$	$49.2±1.63^{++}$	$84.5±2.40^{++}$
**$NHFH_{100}$**	$6.66±0.38^{++}$	$1.11±0.008^{++}$	$4.05±0.70^{++}$	$54.5±1.63^{++}$	$87.5±3.49^{++}$
**$NHFH_{200}$**	$6.75±0.40^{++}$	$1.09±0.006^{++}$	$2.9±0.66^{++}$	$46.34±2.07^{++}$	$82.6±1.93^{++}$

Table S3: Nephroprotective effects of *Fraxinus hookeri* different fractions on serum profile levels including serum protein, globulin, serum urea, albumin, and serum nitrite in rats, with Mean ± SD shown for every group (n = 6). ++ indicate significance from the CCl_4 group at P < 0.05 and probability level is P < 0.01. ** indicate significance from the Control group at P < 0.05 and probability level is P < 0.01.

Groups	Serum protein (mg/dL)	Serum Urea (mg/dL)	Serum nitrite (µM/mL)	Albumin (mg/dL)	Globulin (mg/dL)
**Control**	$34.69 \pm 1.65^{++}$	$39.7 \pm 2.01^{++}$	$41.45 \pm 2.33^{++}$	$25.56 \pm 0.99^{++}$	$41.45 \pm 2.20^{++}$
**DMSO**	$35.18 \pm 1.41^{++}$	$41.1 \pm 1.63^{++}$	$44.26 \pm 1.99^{++}$	$24.14 \pm 0.86^{++}$	$42.33 \pm 1.63^{++}$
**CCl4**	$22.88 \pm 1.45^{**}$	$72.2 \pm 2.44^{**}$	$84.18 \pm 2.25^{**}$	$9.69 \pm 0.60^{**}$	$25.78 \pm 1.32^{**}$
**Rutin**	$33.95 \pm 1.61^{++}$	$46.02 \pm 2.94^{++}$	$52.54 \pm 2.22^{++}$	$21.89 \pm 0.97^{++}$	$39.22 \pm 1.64^{++}$
**$MEFH_{100}$**	$25.25 \pm 1.81^{++}$	$53.1 \pm 2.44^{++}$	$62.22 \pm 2.69^{++}$	$14.67 \pm 0.89^{++}$	$30.14 \pm 1.68^{++}$
**$MEFH_{200}$**	$28.42 \pm 2.002^{++}$	$49.09 \pm 1.63^{++}$	$55.89 \pm 2.36^{++}$	$18.76 \pm 0.65^{++}$	$36.02 \pm 1.60^{++}$
**$NHFH_{100}$**	$26.67 \pm 1.94^{++}$	$51.28 \pm 1.70^{++}$	$58.23 \pm 2.49^{++}$	$16.05 \pm 0.75^{++}$	$33.53 \pm 1.76^{++}$
**$NHFH_{200}$**	$30.69 \pm 1.69^{++}$	$47.87 \pm 1.26^{++}$	$53.6 \pm 2.51^{++}$	$20.98 \pm 0.79^{++}$	$37.69 \pm 1.68^{++}$

Table S4: Nephroprotective effects of *Fraxinus hookeri* different fractions on serum profile levels including direct bilirubin, total bilirubin, creatinine, urobilinogen, and creatinine clearance in rats, with Mean ± SD shown for every group (n = 6). ++ indicate significance from the CCl4 group at P < 0.05 and probability level is P < 0.01. ** indicate significance from the Control group at P < 0.05 and probability level is P < 0.01.

Groups	Direct Bilirubin (mg/dL)	Total bilirubin (mg/dL)	creatinine (mg/dL)	Creatinine Clearance (mL/min)	Urobilinogen (mg/dL)
**Control**	$1.69 \pm 0.12^{++}$	$3.87 \pm 0.20^{++}$	$40.9 \pm 1.79^{++}$	$0.84 \pm 0.03^{++}$	$14.33 \pm 1.22^{++}$
**DMSO**	$1.71 \pm 0.13^{++}$	$4.05 \pm 0.25^{++}$	$41.69 \pm 2.22^{++}$	$0.86 \pm 0.02^{++}$	$15.09 \pm 1.36^{++}$
**CCl4**	$2.45 \pm 0.11^{**}$	$4.69 \pm 0.24^{**}$	$79.87 \pm 1.98^{**}$	$0.56 \pm 0.03^{**}$	$31.74 \pm 1.27^{**}$
**Rutin**	$1.83 \pm 0.13^{++}$	$4.18 \pm 0.22^{++}$	$48.12 \pm 2.13^{++}$	$0.76 \pm 0.02^{++}$	$18.04 \pm 0.81^{++}$
**$MEFH_{100}$**	$2.02 \pm 0.10^{++}$	$4.42 \pm 0.21^{++}$	$61.11 \pm 2.16^{++}$	$0.61 \pm 0.01^{++}$	$24.02 \pm 1.42^{++}$
**$MEFH_{200}$**	$1.91 \pm 0.08^{++}$	$4.27 \pm 0.19^{++}$	$53.34 \pm 1.89^{++}$	$0.69 \pm 0.03^{++}$	$20.33 \pm 0.90^{++}$
**$NHFH_{100}$**	$1.94 \pm 0.11^{++}$	$4.34 \pm 0.15^{++}$	$56.28 \pm 1.90^{++}$	$0.64 \pm 0.04^{++}$	$22.55 \pm 1.22^{++}$
**$NHFH_{200}$**	$1.86 \pm 0.08^{++}$	$4.23 \pm 0.18^{++}$	$52.33 \pm 1.92^{++}$	$0.72 \pm 0.03^{++}$	$18.96 \pm 1.28^{++}$

Table S5: Evaluation of the nephroprotective effects of *Fraxinus hookeri* different fractions on the enzymatic antioxidant levels and %DNA fragmentation in rats, with Mean ± SD shown for every group (n = 6). ++ indicate significance from the CCl_4 group at $P < 0.05$ and probability level is $P < 0.01$. ** indicate significance from the Control group at $P < 0.05$ and probability level is $P < 0.01$.

Groups	CAT (U/min)	POD (U/min)	SOD (U/mg protein)	TBARS (nM/min/mg protein)	%DNA fragmentation
**Control**	5.74±0.54^{++}	13.26±0.63^{++}	20.63±0.65^{++}	26.63±2.20^{++}	9.95±0.91^{++}
**DMSO**	5.6±0.45^{++}	13.23±0.57^{++}	20.26±0.91^{++}	26.21±1.9^{++}	8.64±1.24^{++}
**CCl4**	2.84±0.47**	6.56±0.80**	9.93±1.02**	38.50±1.25**	35.66±1.49**
**Rutin**	4.96±0.49^{++}	12.5±0.77^{++}	18.63±1.59^{++}	28.53±2.47^{++}	12.31±0.46^{++}
**$MEFH_{100}$**	3.06±0.54^{++}	9.16±0.76^{++}	14.93±1.46^{++}	33.33±1.10^{++}	20.36±1.23^{++}
**$MEFH_{200}$**	3.86±0.32^{++}	10.88±0.85^{++}	17.26±0.89^{++}	30.50±2.14^{++}	15.99±1.62^{++}
**$NHFH_{100}$**	3.26±0.55^{++}	9.86±0.85^{++}	15.96±1.11^{++}	32.06±1.40^{++}	18.03±1.67^{++}
**$NHFH_{200}$**	4.36±0.39^{++}	11.95±0.4^{++}	17.83±0.70^{++}	29.46±1.56^{++}	13.98±0.81^{++}
